# Reducing the risk of non-sterility of aseptic handling in hospital pharmacies, part A: risk assessment

**DOI:** 10.1136/ejhpharm-2019-002178

**Published:** 2020-05-08

**Authors:** Frits A Boom, Judith M Ris, Tjitske Veenbaas, Paul P H Le Brun, Daan Touw

**Affiliations:** 1 Zaans Medical Centre, Zaandam, Noord-Holland 1502DV, The Netherlands; 2 Albert Schweitzer Hospital Location Dordwijk, Dordrecht, Zuid-Holland 3318 AT, The Netherlands; 3 Department of Clinical Pharmacy and Toxicology, Leiden University Medical Centre, Leiden, The Netherlands; 4 Clinical Pharmacy, UMCG, Groningen, The Netherlands

**Keywords:** aseptic preparation, audit, self-inspection, compounding (individualised preparation), disinfection, Good Manufacturing Practice (GMP), manufacturing, small scale, protocols & guidelines, reconstitution, risk management, validation preparation process

## Abstract

**Objectives:**

To determine prospectively the sources of risk of non-sterility during aseptic handling and to quantify the risks of each of these sources.

**Methods:**

A risk assessment (RA) of non-sterility according to Failure Mode and Effect Analysis was executed by a multidisciplinary team of (hospital) pharmacists and technicians, a consultant experienced in aseptic processing and an independent facilitator. The team determined the sources of risk of non-sterility, a 5 point scale for severity, occurrence and detection, and risk acceptance levels. Input about general applied risk reduction was collected by audits in 10 hospital pharmacies. The results of these audits were used for determining the remaining risks. The results, as well as scientific information and the experience of the team members, was used to determine scores for severity, occurrence and detection.

**Results:**

Multiplying the scores for severity, occurrence and detection results in the risk prioritisation number (RPN) which is a relative value of the remaining risks of non-sterility for each source. Incorrect disinfection techniques of non-sterile materials and the chances of touching critical spots were estimated as the greatest risks. The risk of non-sterility via the airborne route was low. RPN values were helpful in prioritising measures for additional risk reduction (this will be described in an accompanying article).

**Conclusion:**

The RA, described here, was a systematic survey related to all sources of risk of non-sterility during aseptic handling. The determined RPN values were helpful in prioritising measures for additional risk reduction.

## Introduction

To improve patient safety in hospitals, the preparation of ready to administer injections and infusions is becoming more centralised in hospital pharmacies. In Europe, this preparation process is called ‘aseptic handling’ or ‘aseptic preparation’, and in the USA the term ‘compounding sterile preparations’ is used.^1–3^ Aseptic handling is defined as a procedure to enable sterile medicinal products to be made ready to administer, using closed systems.^1^ Throughout this article, we will use this term, because the definition contains the words ‘closed systems’, which is an essential aspect of making sterile products ready to administer.

Professionals, as well as authorities, have formulated standards to improve the quality of aseptic handling.^1–6^ However, infections related to aseptic handling do occur and can have marked consequences, especially when the products are distributed from one centre to more hospitals.^7^

The operator contributes by far the greatest risk to microbial contamination during aseptic handling.^8 9^ Protective clothing can reduce contamination, but being trained in aseptic techniques is equal important. The contribution of the environment as a source of risk for contamination is reduced by working in a laminar airflow cabinet (LAF), safety cabinet (SC) or isolator, and the risks from materials used during aseptic handling are reduced by disinfection by wiping with alcohol impregnated wipes.^10^ It is not known which of these sources is the most important. The same is true for measures to reduce the risk of microbiological contamination: which is most effective? Answering these questions can be done with a risk assessment (RA) where, in a systematic process, risks are analysed and evaluated to support risk management.^11 12^

A risk is defined as the combination of the probability of occurrence of harm and the severity of that harm.^11^ The probability of occurrence depends on the occurrence itself and the chances of detecting it. During an RA, risk is quantified by determining values for severity, occurrence and detection. The first step is to pose the question, ‘What can go wrong’, and to make a list of the potential process failures (risk identification). The second step is risk analysis: linking the likelihood of occurrence of a failure with the ability to detect it and the severity of the unwanted event. Risk is quantified either qualitatively (high/medium/low) or semi-quantitatively by calculating a risk prioritisation number (RPN), which is obtained by multiplying the scores for severity, occurrence and detection. The third step is risk evaluation, comparing potential process failures to pre-established risk acceptance criteria. In the last step, risk control (RC), risks above the acceptance criteria are proactively reduced and/or detection is improved. However, the effort made to reduce the amount of risk should be proportional to the impact of the risk.^12^ Certain risks may be accepted as a consequence.

There are many risk management tools available.^11^ Failure Mode and Effect Analysis (FMEA) is often used in the field of pharmacy. Preconditions include using scientific information, expert opinions and rigorous thinking in a multidisciplinary team.^12^

In this study, we describe an RA for the risk of non-sterility in aseptic handling, which can be used to quantify and prioritise risks and to initiate additional risk reduction measures. Definitions of terms, which are less common, are given in online supplementary file 1. In part B of this series of articles, additional risk reduction is worked out in an RC model.^13^ Supplementary investigations, to make RC more robust, are also described in part B.^13^

10.1136/ejhpharm-2019-002178.supp1 Supplementary data

There is only limited experience with isolators in The Netherlands. Therefore, we restricted both studies (parts A and B) to aseptic handling done in an LAF or SC.

## Materials and methods

We developed a risk management tool for aseptic handling from the example, ‘Risk assessment of aseptic filling’, described in the Parenteral Drug Association Technical Report 44.^12^ The model is a combination of RA according to FMEA and an RC strategy. Risk identification, determining the remaining risks and the RA were executed by a multidisciplinary team of (hospital) pharmacists and technicians, a consultant experienced in aseptic processing and an independent facilitator.

### Risk identification

‘What might go wrong’ in aseptic handling within the scope of this RA is non-sterility of the product that is prepared. Sterility failure is difficult to detect.^14^ Therefore, sources of risk that could result in microbiological contamination of the product were listed to identify the risk of non-sterility.

#### Chance of contamination via the airborne route

Whyte gives a formula for settling of particles from the air onto a surface^15^:

(1) $$Numberofparticlesdeposited=0.0032d^{2}\times C\times A_{n}\times t$$

where d=particle diameter, C=particle concentration, A_n_=surface in cm^2^ and t=time in minutes particles deposed. If only A_n_ and t change and C is restricted to viable particles only, the following formula can be derived from equation (1) for comparing viable particle (cfu) deposition on different surfaces at different deposition times:

(2) $$Numberofcfudepositonagivenobject=X\times t_{1}/t_{2}\times A_{1}/A_{2}$$

where X=number of cfu on a settle plate, t_1_=time an open vial or ampoule, or a given object remains in an environment (min), t_2_=sedimentation time of a settle plate in the same environment, A_1_=opening of a vial or an ampoule, or the cross section of a given object in cm^2^ and A_2_=surface of a settle plate (Ø 90 mm, 64 cm^2^)

#### Audits and remaining risks

The chapter ‘Aseptic handling’ of the Good Manufacturing Practice (GMP)-hospital pharmacy was used as a starting point.^6^ This means working in a disinfected LAF or SC, located in an EU grade D (or better) background room, surface disinfection of materials used in the LAF/SC, qualified operators wearing clean room clothing and sterile gloves, and controls such as microbiological monitoring and broth simulations.^8^

How the ‘Aseptic handling’ (GMP-hospital pharmacy) was put into practice, and which risks remained, was audited in 10 hospital pharmacies (two academic, five top clinical and three regional). Three of the pharmacies used an LAF (cross flow) and seven used an SC (down flow) as a work environment. The audits were performed by an experienced technician and a hospital pharmacist involved in aseptic handling. The results were recorded in audit reports which were used by the multidisciplinary team to determine the remaining risks.

### Risk assessment

For risk analysis, a 5 point scale for severity, occurrence and detection was used. For risk evaluation, risk acceptance values were determined. The results of the audits were used by the multidisciplinary team to determine the remaining risk for each risk source. Subsequently, using available scientific information and the experience of the team members, the corresponding scores for severity, occurrence and detection were determined.

## Results

### Risk identification

Sources of risk resulting in microbiological contamination of the product were the LAF or SC itself and all items introduced into these cabinets, including the operator. The sources of risk are summarised in table 1, divided into three areas: work area (A–C), transfer of materials (D, E) and operator (F–H).

**Table 1:** Sources of risk of non-sterility during aseptic handling

Code	Description
A	Air in LAF/SC
B	Worktop LAF/SC
C	Wall and ceiling LAF/SC
D1	Materials with a sterile surface (tubes, syringes, needles, infusion bags, etc)*
D2	Critical spots† such as the opening of tubes, syringe tips, needles, septa of infusion bags
E1	Materials and equipment with a non-sterile surface (ampoules, vials, bottles, etc)*
E2	Critical spots† such as vial stoppers, ampoule necks
F	Operators' hands
G	Operators' forearms
H	Working procedure

*For an extensive explanation see Boom *et al.* 22

†A definition of ‘critical spots’ is given in online supplementary file 1.

LAF, laminar airflow cabinet; SC, safety cabinet.

### Chance of contamination via the airborne route

Deposition in an open ampoule in grade A air: The number of deposed microorganisms in 240 min (t_2_) on a settle plate (A_2_, 64 cm^2^), in an LAF/SC in Dutch hospital pharmacies, was <0.1 cfu.^16^ The hole of an open ampoule is approximately 3.14×10^-2^ cm^2^ (A_1_). Using equation (2), the number of cfu which can contaminate a sterile solution inside the ampoule in 5 min (t_1_) was, at most, 0.1×5/240×3.14×10^-2^/64=1×10^-6^.

Deposition on the surface of disinfected materials outside the LAF/SC: The number of deposed microorganisms in 240 min (t_2_) on a settle plate (A_2_, 64 cm^2^), in a grade C and grade D environment in Dutch hospital pharmacies, were about 5 and 10 cfu, respectively.^16^ Using equation (2), the number of cfu deposits in 5 min (t_1_) on a 100 mL glass bottle (A_1_=20 cm^2^) were 5×5/240×20/64=0.033 cfu (grade C) or 10×5/240×20/64=0.065 cfu (grade D).

### Audits and remaining risks

**Figure 1:**
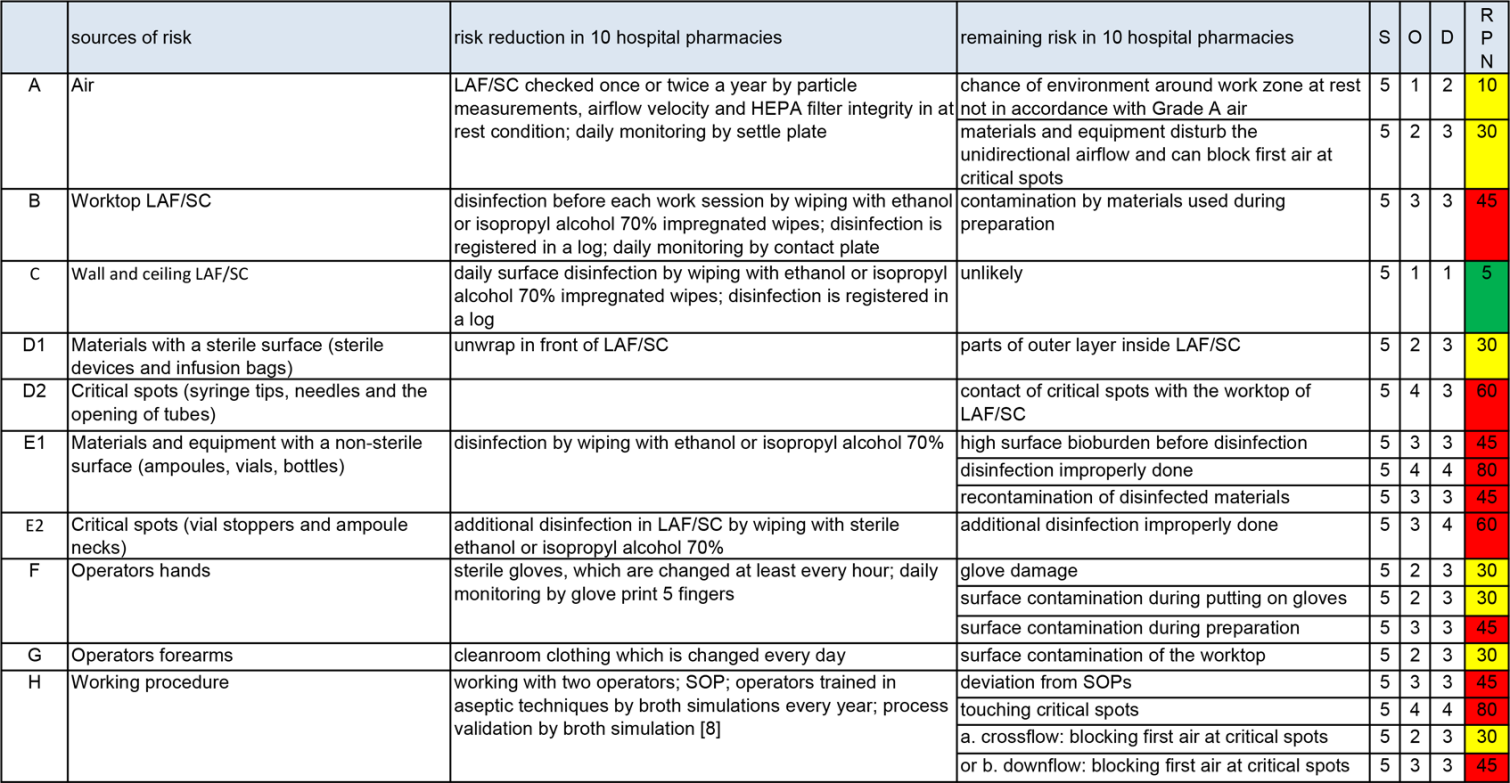
Risk assessment of non-sterility for aseptic handling. D, detection; LAF, laminar airflow cabinet; O, occurrence; RPN, risk prioritisation number; S, severity; SC, safety cabinet; SOPs, standard operating procedures.

In all hospital pharmacies audited, ‘Aseptic handling’ of the Dutch GMP-hospital pharmacy was fully implemented.^6^ If a risk reduction measure was applied in at least 8 out of the 10 audited hospital pharmacies, the multidisciplinary team listed this in figure 1 column ‘risk reduction in 10 hospital pharmacies’.

### Risk assessment

#### Risk analyses

The 5 point scale for occurrence and detection is shown in table 2.

**Table 2:** Five point scale for occurrence and detection

Occurrence		Detection	
1	Low	1	Certainly discovered
2	Probably low	2	Probably discovered
3	Medium	3	Mean chance of discovering
4	Probably high	4	Low chance of discovering
5	High	5	Not discovered

Events resulting in, or contributing to, the loss of sterility will always be scored high because of the direct and potentially severe impact on the patient. Therefore, in common with the Technical Report 44 model, severity will always be scored as the maximum number of points (a score of 5 in our study).^12^

The audits in the 10 hospital pharmacies indicated that a considerable number of risk reducing measures had already been applied in daily practice. Therefore, a score of 5 for occurrence and detection was not used in the RA. Occurrence of non-sterility has to be considered as a relative value and cannot be expressed as once a day, once a week, etc. Detection is a matter of measuring (eg, monitoring), observation (eg, auditing) and registration (eg, writing down in a log).

#### Risk acceptance

**Figure 2:**
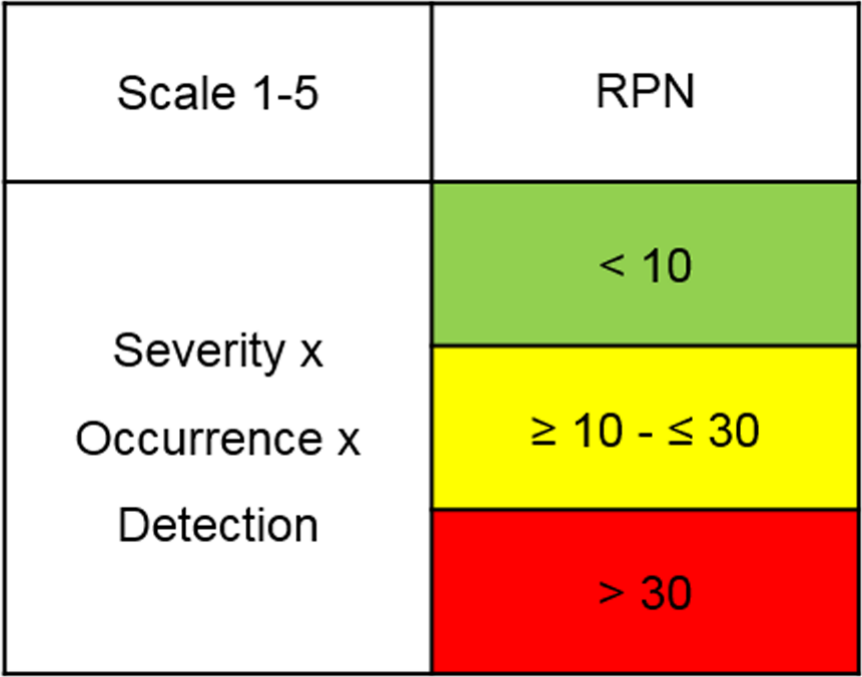
Risk acceptance values. Green=safe, no action; yellow=nearly safe, possible action; red=not safe, action. RPN, risk prioritisation number.

The risk acceptance values are described in figure 2. The minimum RPN was 5 because, as mentioned previously, severity is always scored 5. An RPN <10 (green) implies safe (no action), ≥10 and ≤30 implies nearly safe (yellow, possible action) and >30 implies not safe (red, action).

##### Risk scores

Figure 1 shows the complete RA of aseptic handling. The columns ‘remaining risk in 10 hospital pharmacies’ and the values for occurrence and detection were the results of the discussion in the multidisciplinary team. The calculated RPNs showed that only one risk source was considered safe (green); seven were yellow and nine were red.

## Discussion

Sivika-Peltonen *et al* and Austin *et al* published a systematic review on incorrect aseptic techniques during aseptic handling.^9 17^ Both studies are informative on the possible risks of non-sterility, but the primary object was comparing aseptic handling on the ward with aseptic handling in the pharmacy.

Our study was restricted to hospital pharmacies only. The objectives were to determine all sources of risk of non-sterility, to describe common risk reduction measures and to determine remaining risks. As shown in figure 1, FMEA can facilitate this process. The calculated RPN values can help in prioritising the remaining risks and defining additional risk reducing measures (the latter will be discussed in part B of our series of articles^13^). The chapter ‘Aseptic handling’ of the GMP-hospital pharmacy was used as a starting point in this study.^6^ How this was put into practice is shown in figure 1 (column ‘risk reduction in 10 hospital pharmacies’). In view of the diversity of the hospital pharmacies audited (small, big, academic) we believe these results can be expected in many hospital pharmacies in The Netherlands. Consequently, the items in figure 1 ‘remaining risk’, can also be expected. Background information on ‘remaining risks’ as well as scores for occurrence, detection and the accompanying RPN values will be discussed below.

### Work area

#### Air

The risk of non-sterility via the airborne route was low because aseptic handling was done using closed systems. Different studies have confirmed this.^18–20^ Using ampoules can be regarded, in principle, as a closed procedure^2^ (see 'Chance of contamination via the airborne route’).

The low risk of non-sterility via the airborne route should not lead to reduced attention to the air quality inside the LAF/SC because the number of particles (marker for microorganisms) at critical spots must not exceed the limits for grade A air. Therefore, dysfunction of the LAF/SC and disturbance of the unidirectional airflow by materials and equipment are remaining risks.

All LAF and SC are checked once or twice a year by certified companies. The question arises as to what to do if a defect is found during that check, because it is unclear when the defect appeared; theoretically, it could be any time after the previous check. In part B of this series of articles, investigations on the chances of defects in the LAF/SC are described.^13^

The consequences of disturbing the unidirectional flow by materials and equipment, and by the hands and forearms of the operator, are blocking of first air at critical spots, such as syringe tips, needles, vial stoppers and open ampoules. Airflow visualisation (smoke studies) can be used to find the right position for materials in the LAF/SC as well as the correct way of working.^21^ This will also be described in part B.^13^

Considering the low risk of non-sterility via the airborne route, the team estimated the occurrence at ‘low’ (1 point) for the environment around the work zone and ‘probably low’ (2 points) for disturbing unidirectional flow (figure 1). Additional studies are needed to decrease the values for detection. The RPN for the two remaining risks were 15 and 30. For moving parts (materials) and personnel (hands and forearm), the chances of disturbing unidirectional flow and blocking first air on critical spots are discussed below.

#### Worktop LAF/SC

Microorganisms can be dragged onto the worktop by materials (see 'Transfer of materials'). If the worktop is not regularly disinfected, the operators’ gloved hands can be contaminated by the worktop, which is a substantial risk of non-sterility (see 'Operator'). Worktop disinfection before each new prepared dosage form is not common practice. Therefore, the team estimated the RPN for the worktop at 45 (figure 1).

As mentioned in the introduction and in figure 1, surface disinfection in Dutch hospital pharmacies is executed by wiping with alcohol impregnated wipes. This means that microorganisms are inactivated by the disinfectant and also removed mechanically.^10^ Wiping with alcohol impregnated wipes also cleans the surface.^2^ This makes separate worktop cleaning only necessary if the worktop is seriously smudged.

#### Wall and ceiling LAF/SC

The team concluded that the risk reduction measures, found during the audits and mentioned in figure 1, were sufficient (RPN=5).

### Transfer of materials

#### Materials with a sterile surface

Materials with a sterile surface (D1), such as sterile medical devices and infusion bags, are wrapped in one or more layers and sterilised. They are partly unwrapped in front of the LAF/SC by a secondary operator and presented to the primary operator (primary and secondary operator, see ‘Working procedures’).^22^ In contrast with unwrapping in front of the LAF/SC, presenting is not common practice, which means that parts of the non-sterile outer layer will come inside the LAF/SC and can contaminate the worktop. Therefore, the team estimated the RPN at 30 (figure 1).

Critical spots of materials with a sterile surface (D 2) are syringe tips, needles and the opening of tubes. They must be kept sterile at all times. Non-touch working, to prevent contact of critical spots with non-sterile surfaces, can be improved. Therefore, the team estimated the RPN at 60.

#### Materials with a non-sterile surface

Materials with a non-sterile surface, such as ampoules, vials and bottles (E1), must be disinfected before being transferred into the LAF/SC. A low surface bioburden before disinfection, as well as an effective disinfection procedure, is important.^10 22^ This is not common practice, as well as measures, to prevent recontamination after disinfection. Therefore, the tree possibilities of bringing microorganisms by non-sterile materials into the LAF/SC are a real risk, which are expressed in relatively high RPN values of 45, 80 and 45 respectively (figure 1).

Materials are disinfected in the background area (EU grade D or better) and placed there before transfer into the LAF/SC. The results for deposition of cfu on disinfected materials (see ‘Chance of contamination via the airborne route’) made clear that the risk of additional contamination in the background area was low.

**Figure 3:**
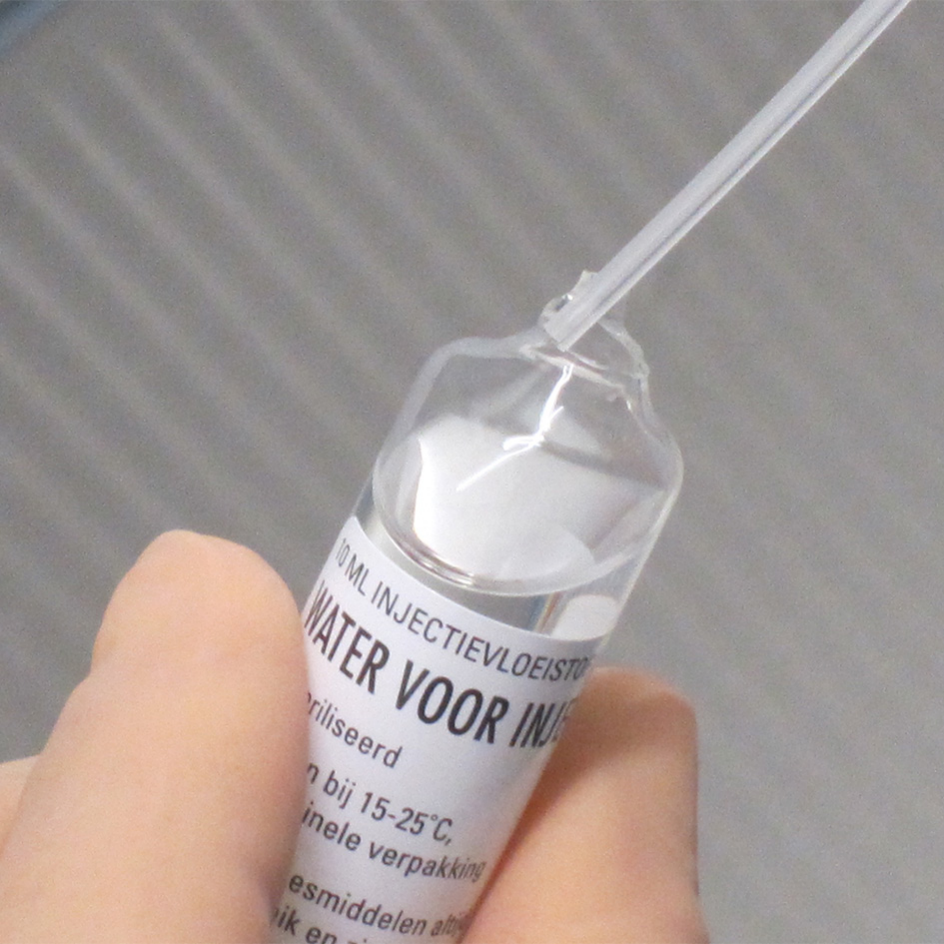
Touching the neck of an open ampoule by a plastic needle.

Touching the neck of an open ampoule by a needle can happen easily (figure 3). Therefore, stoppers and ampoule necks are critical spots (E2). To prevent microbial contamination, these spots need additional disinfection inside the LAF/SC.^23^ This is common practice, but the method of disinfection can be improved by more thorough wiping and a longer contact time (at least 30 s). Because of the high risk of contaminated critical spots (contact with the needle or spike and therefore with the sterile fluid) the team estimated the RPN at 60 (figure 1).

### Operator

#### Operators’ hands

Aseptic handling is done by manual procedures, which means that the operators’ hands will touch many surfaces in the LAF/SC and will come close to critical spots. Wearing sterile gloves and keeping the surface bioburden of these gloves as low as possible is therefore very important.^24^ The risk of product contamination by gloves can be divided into glove damage, contamination when putting the gloves on and contamination during preparation (figure 1).

Gloves can be thin; this makes them sensitive to damage (tears or gaps). The chances of contamination by non-visible damage (pinholes) are low.^25^ Visible damage must be prevented by checking the integrity of the gloves regularly. This is not general practice and therefore the team gave the remaining risk an RPN of 30.

In general, there is a lack of information in standard operating procedures (SOP) about the correct way of putting on gloves. Consequently, the chances of contamination of the sterile surface of the gloves when putting them on are real (RPN of 30).

Non-sterile materials can drag microorganisms inside the LAF/SC. During preparation, many of these materials are held with the gloved hands of the operator and can contaminate the sterile surface of the gloves. As described in a previous study, disinfection of non-sterile materials in The Netherlands can be improved.^10^ Regular glove disinfection is not common practice. Both shortcomings were also found in the audited hospital pharmacies. Therefore, contamination of gloves during preparation was a serious risk (RPN of 45).

#### Operators’ forearms

The operators’ forearms (risk source G) can be in the LAF/SC. Cleanroom clothing, covering the forearm, is not sterile or will not stay sterile, and therefore can contaminate the worktop. In only one of the audited hospital pharmacies was the risk of non-sterility from this risk source diminished by wearing sterile sleeves. Therefore, the team estimated the RPN at 30 (figure 1). Blocking first air on critical spots, an other remaining risk of the forearm, is discussed below.

#### Working procedures

Working with two operators is strongly advised.^26^ The primary operator performs all tasks inside the LAF/SC and the secondary operator supports the transfer of materials into the LAF/SC and carries out all the activities outside the LAF/SC, for example, collecting and disinfecting materials and labelling after preparation. Working with two operators was common practice in 8 of the 10 hospital pharmacies audited.

Microbiological controls can demonstrate the quality of aseptic processing but these controls are not sensitive enough to guarantee the absence of incorrect working procedures.^8 12^ Therefore, regular auditing of each operator is an important additional tool in risk reduction. However, auditing is not a general practice (a procedure is described in part B of this series of articles^13^).

The remaining risks of working procedures are deviations from SOP, touching critical spots and blocking first air (figure 1). The way in which working procedures are specified in SOP as well as working discipline, can lead to deviations from SOP. Both can be improved. Therefore, the RPN for deviations from SOP was estimated at 45.

Touching needles or spikes onto non-sterile surfaces is a great risk of non-sterility in aseptic handling.^20 23 24^ The same is true for touching other critical spots, such as vial stoppers and ampoule necks. Because of the low sensitivity of microbiological controls (see above), non-touch working is an important topic for additional controls, such as auditing. However, this was common practice in only two of the hospital pharmacies audited. Therefore, the RPN for touching critical spots was estimated at 80.

The audits showed that working in first air needs more attention. Regarding working with closed systems, the consequences of blocking first air at critical spots by moving parts (materials) and personnel (hands and forearm) was lower compared with touching critical spots. Blocking first air in downflow (SC), compared with crossflow (LAF), occurs more easily.^13^ Therefore, the team estimated the occurrence in crossflow at 2 points and in downflow at 3 points, making the RPN for this remaining risk 30 and 45, respectively (figure 1).

### Risk control

In risk control, additional measures are implemented to reduce the risks of non-sterility to an acceptable level (if possible to a safe (green) RPN score).^11 12^ Additional investigations are necessary to work out the RC for all sources of risk. These investigations, as well as an RC model with RPN calculations derived from figure 1, are described in part B of this series of articles.^13^

## Conclusion

The RA, described here, was a systematic survey, related to all sources of risk of non-sterility during aseptic handling. The RPN values were helpful in prioritising measures for additional risk reduction. Incorrect disinfection techniques of non-sterile materials and the chances of touching critical spots were estimated as the greatest risks. The risk of non-sterility via the airborne route was low.

Additional studies on the chances of defects in the LAF/SC and disturbing unidirectional flow inside the LAF/SC, as well as auditing during aseptic handling, are necessary to elucidate risk reduction. These studies, as well as a model for risk control, will be described in an accompanying article.

**Key messages**

**What is already known on this subject**

- Aseptic handling should be executed with aseptic precautions in a laminar airflow cabinet, safety cabinet or isolator
- The operator is the highest source of risk of non-sterility

**What this study adds**

- A method for quantifying and ranking the different sources of risk of non-sterility during aseptic handling was established
- The gloved hands of the operator and the materials used during aseptic handling (ampoules, vials, sterile medical devices) are important sources of risk of non-sterility
- The risk of non-sterility via the airborne route during aseptic handling is low

## Data Availability

All data relevant to the study are included in the article or uploaded as supplementary information. An Excel file with the data for figure 2 is available.
